# The Roles of microRNAs in Regulating the Expression of PD-1/PD-L1 Immune Checkpoint

**DOI:** 10.3390/ijms18122540

**Published:** 2017-11-27

**Authors:** Qingshui Wang, Wei Lin, Xiaoqiong Tang, Suhuan Li, Libin Guo, Yao Lin, Hang Fai Kwok

**Affiliations:** 1 Provincial University Key Laboratory of Cellular Stress Response and Metabolic Regulation, College of Life Sciences, Fujian Normal University, Fuzhou 350117, China; wangqingshui@fjnu.edu.cn (Q.W.); txq242347@gmail.com (X.T.); lisuhuan06@gmail.com (S.L.); 2 Faculty of Health Sciences, University of Macau, Avenida de Universidade, Taipa, Macau SAR, China; yb67625@umac.mo; 3 Academy of Integrative Medicine, Fujian University of Traditional Chinese Medicine, Fuzhou 350112, Fujian, China; 2012001@fjtcm.edu.cn

**Keywords:** programmed death-ligand 1 (PD-L1), programmed death 1(PD-1), microRNAs, immune checkpoint

## Abstract

Engagement of programmed death-ligand 1 (PD-L1) with its receptor programmed death 1 (PD-1) on T cells has been speculated to play a major role in suppressing the immune system, which helps tumor cells evade anti-tumor immunity. With the development of whole genome sequencing technologies, microRNAs have gained more attention as an important new layer of molecular regulation. Recent studies have revealed that altered expression of microRNAs play a pivotal role in immune checkpoint and various cellular processes in cancer. In this review, we focused on the latest progress about microRNAs research which involves the regulation of PD-1/PD-L1 immune checkpoint.

## 1. Introduction

The most exciting event in the past five years within anti-cancer battle field is the emergence of immune checkpoint therapy, which now cuts a clear path out and joins the battle array of surgery, radiation, chemotherapy, and targeted therapy as a strong enforcement of cancer therapy. Immuno checkpoint molecules such as cytotoxic T lymphocyte-associated antigen-4 (CTLA-4, CD152) and programmed cell death protein 1 (PD-1) are membrane proteins residing on T lymphocytes and deliver negative signals to limit T lymphocyte mediated immune responses. These checkpoint molecules belong to a family of cell signaling molecules named co-stimulatory molecules that regulates T lymphocyte functions positively or negatively along with T cell receptors. PD-1/Programmed death-ligand (PD-L) pathway consists of PD-1 and its two ligands. PD-1 is inducibly expressed on T cells, B cells, Natural Killer T cells (NKT cells), and monocytes upon activation. Programmed death-ligand 1 (PD-L1) is expressed on antigen presenting cells (B cells, dendritic cells, macrophages), cultured bone marrow derived mast cells and T cells. PD-L1 is also expressed on non-hematopoietic cells such as astrocytes, pancreatic islet cells, keratinocytes and vascular endothelial cells, etc. The program death-ligand 2 (PD-L2) is only expressed inducibly on dendritic cells (DCs), macrophages, and bone marrow derived cultured mast cells. Under normal physiological conditions, PD-L1 mRNA was found expressed broadly in various tissues, but PD-L1 protein is only found on a few tissues such as tonsil, a small fraction of macrophage-like cells in lung and liver, and placenta, suggesting PD-L1 mRNA is tightly regulated by post-transcriptional machinery [1,2]. In contrast, PD-L1 protein was found expressed on various human cancer cells. Although many cytokines are indicated to play roles in inducing or maintaining PD-L1 expression, both in vitro and in vivo studies have proved that expression of PD-L1 protein in antigen presenting cells (APCs), regulatory T lymphocytes, and cancer cells strongly relied on the existence of interferon-gamma (IFN-γ). Inflammatory cells, especially activated T lymphocytes are usually considered the major source of IFN-γ. It is proposed that PD-L1 is up-regulated in response to inflammation and suppresses excessive immune responses which may cause unnecessary tissue injury. Tumor cells that arise from normal cells adopt this mechanism to evade tumor immunity [3,4].

Several signaling pathways were reported to be involved in the regulation of PD-L1 in multiple types of tumors. The two major signaling pathways are Just Another Kinase (JAK)/Signal transducers and activators of transcription (STAT)/phosphatidylinositol 3-kinase (PI3K)/protein kinase B (AKT)/methyl ethyl ketone (MEK)/extracellular regulated protein kinases (ERK) and JAK/src homology 2-containing tyrosine phosphatase (SHP2)/rat sarcoma (RAS)/rapidly accelerated fibrosarcoma (RAF)/MEK/ERK pathways. Clearly the MEK/ERK kinases involved in both pathways possess critical roles for IFN-γ induced expression of PD-L1. For example, PD-L1 expression induced by IFN-γ was found blocked by inhibition of MEK/ERK pathway using U0126 (inhibitor of MEK1/2) in multiple myeloma (MM) plasma cells and hepatic stellate cells (HSCs) [5,6].

PD-L1 was also regulated by phosphatase and tensin homolog deleted on chromosome ten (PTEN)/PI3K/AKT/mammalian target of rapamycin (mTOR) pathway. Loss of PTEN promoted cell proliferation, cell invasion and significant increase in the levels of phospho-AKT and phospho-mTOR, resulting in enhanced protein translation of PD-L1 in human glioma [7]. In addition, interferon regulatory factor 1 (IRF1) is a downstream target of STAT1and the critical transcription factor for PD-L1 expression. Knockdown of IRF1 using siRNA greatly decreased both the constitutive and IFN-γ-induced expression of PD-L1 inA549 cells [8]. Moreover, it can be speculated that IFN-γ receptor (IFNGR) modulating the expression and activation of various signaling molecules mentioned above are probably also involved in the regulation of PD-L1 expression.

**Figure 1:**
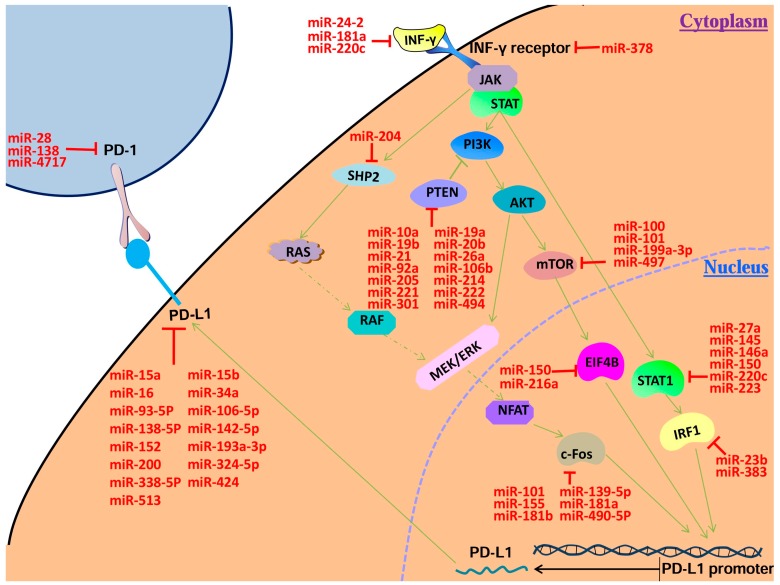
Induction of PD-L1 by IFN-γ signaling pathway and microRNAs target the key genes in the pathway. The green arrow means stimulatory modification, the red “T” symbol means inhibitory modification.

Analysis of human gene transcriptome shows that only 1 to 2 percent of genome sequences have protein-coding capacity, suggesting that there may be a large number of non-coding RNAs [9]. MicroRNAs are small non-coding RNAs consisting of 20–22 nucleotides involved in crucial biological processes including development, differentiation, apoptosis and proliferation [10]. The action mechanism of microRNAs is a multistep process. The genes encoding microRNAs in the nucleus are transcribed into pri-microRNAs which were cut into pre-microRNAs by DroshaRNase [11]. Under the participation of Ras-related nuclear protein and exportin-5, pre-microRNAs were transported into the cytoplasm to be processed into mature microRNAs by Dicer RNase [12,13]. After processing, the double helix of mature microRNA unwinds and one strand is bound to an RNA-induced silencing complex (RISC) to form an asymmetric RISC assembly. Then the complex binds to the target mRNA. MicroRNA is able to couple with its complementary mRNA, leading to degradation of the coupled mRNA. When microRNA and target mRNA are not fully complementary, microRNA is combined with the 3′-UTR of target mRNA and inhibits the protein translation of the mRNA [14,15]. In the past decades, multiple microRNAs have been found to play important roles in cancer and regulate PD-L1 directly or indirectly (Figure 1 and Table 1) [16]. In this review, we focus on the relationship between the PD-1/PD-L1 pathway and microRNA.

## 2. Current Knowledge on MicroRNAs Involved in PD-1/PD-L1 Regulation

Some microRNAs have been found to directly target the 3′-UTR of PD-1 or PD-L1 mRNA. Some microRNAs may regulate PD-1/PD-L1 indirectly via signaling molecules such as PTEN, IFR-1, and so on. In this section, we will discuss these two groups of microRNAs separately.

### 2.1. MicroRNAs That Regulate PD-1/PD-L1 Directly

#### 2.1.1. MicroRNAs Regulating PD-1 Expression

Results from dual luciferase assays demonstrated that miR-28 could inhibit PD-1 through binding to its 3′-UTR. The expression of PD-1 was attenuated after transfection with miR-28 mimic in B16F10 cells. The ability of miR-28 in regulating T cell exhaustion was also evidenced by the fact that the expression of PD-1, T-cell immunoglobulin domain and mucin domain 3 (TIM3) and B- and T-lymphocyte attenuator (BTLA) of exhausted T cells was increased by the inhibitor of miR-28 [17].

Target binding algorithms predicted that miR-138 could bind PD-1. Transfection of human CD4+ T cells with miR-138 suppressed PD-1 expression in vitro [18]. Moreover, miR-4717 levels in patients with HBV infection were significantly reduced. The single-nucleotide polymorphisms (SNP) rs10204525 (+8669 G/A) in PD-1 gene was revealed to be associated with chronic Hepatitis B Virus (HBV) infection and located in the 3′UTR of PD-1 gene. MiR-4717 decreased PD-1 expression through binding with PD-1 rs10204525 polymorphic site and plays a role in the disease susceptibility of chronic HBV infection [19].

#### 2.1.2. MicroRNAs Regulating PD-L1 Expression

Compared with PD-1, more microRNAs have been found to target PD-L1 using dual luciferase assay (Table 1).

In addition to luciferase experiments, in argonaute 2 Immunoprecipitation (AGO2 IP) experiments, an interaction between the PD-L1 3′UTR and the miR-15a or miR-16 were also discovered. PD-L1 mRNA dropped in malignant pleural mesothelioma cell line (VMC23) after transfection with miR-15a, miR-15b or miR-16 mimic [20]. Furthermore, an inverse correlation between PD-L1 and miR-34a expression in 44 acute myeloid leukemia (AML) samples was observed. Over-expression of miR-34a in HL-60 andKasumi-1 cells reduced the expression of PD-L1 [21]. Besides, expression of PD-L1 was reduced in bulk cancer cells following treatment with miR-93-5p and miR-106b-5p mimics, respectively [22]. To examine the effect of miR-138-5p on endogenous PD-L1 expression, two cell lines with low miR-138-5p expression, colorectal cancer cell lines (HCT116 and SW620), were transfected with miR-138-5p mimics. PD-L1 protein levels were decreased [23]. RT-PCR and Western blot experiments also demonstrated that miR-142-5p can regulate PD-L1 expression by binding to its 3′UTR. Flow cytometry and RT-PCR experiments demonstrated that miR-142-5p overexpression on tumor cells inhibited the expression of PD-L1 in Panc02 cells [24]. MiR-152 in human gastric cancer tissues was significantly lower than that in matched adjacent normal tissues. Furthermore, there was a marked correlation between the levels of miR-152 and PD-L1 mRNA in gastric cancer tissues, due to miR-152 directly bind to PD-L1 3′UTR and inhibited PD-L1 expression [25]. Transfection with miR-193a-3p led to reduction in PD-L1 mRNA expression in VMC23 cells, the low levels of PD-L1 mRNA in H28 cells were further reduced by miR-193a-3p transfection [20]. Inducible or transient miR-200 expression suppressed PD-L1 expression on human mesenchymal lung cancer cell lines H157, H1155, H1299, and H460 [26]. Dendritic cells expressing miR-324-5p or miR-338-5p displayed reduced ability to induce the surface expression as well as total protein levels of PD-L1 on *M. bovis* BCG infection [27]. MiR-424 regulates the PD-1/PD-L1 pathways in chemo resistant ovarian cancer through direct binding to PD-L1 3′UTR [28]. The level of miR-513 was reduced in cells after exposure to *C. parvum* and increased the expression of PD-L1 [29].

### 2.2. Potential PD-1/PD-L1 Regulatory microRNAs

In the section, we outline the microRNAs that may affect PD-1/PD-L1 expression via regulation of the related signaling pathways such as IFN-γ/IFNGR/JAK/STAT/PI3K/AKT/MEK/ERK and so on.

#### 2.2.1. MicroRNAs Regulating IFN-γ Expression

The 3′UTR of the human IFN-γ gene is a perfect match for the miR-181a seed sequence. Co-transfection of a vector expressing miR-181a with the luciferase reporter vector containing the wide-type IFN-γ 3′UTR decreased the luciferase activity significantly. Overexpression of miR-181a had no significant effect on the luciferase activity when the reporter contained the mutated 3′UTR of the IFN-γ gene, confirming IFN-γ is directly targeted by miR-181a [30]. IFN-γ is also the target of miR-24-2 and miR-220c. In turn, IFN-γ can induce the expression of miR-24-2 and miR-220c [31].

#### 2.2.2. MicroRNAs Regulating IFNGR Expression

The results of real-time PCR of miR-378 and Western blot analysis of the IFNGR1 protein at different stages of corpus luteum (CL) development showed that mir-378 decreased the expression of IFNGR1 protein but not IFNGR1 mRNA [32], suggesting it may regulate IFNGR1 indirectly.

#### 2.2.3. MicroRNAs Regulating STAT1 Expression

STAT1 was directly regulated by miR-145 detected by luciferase assays [33]. MiR-146a, one of the microRNAs prevalently expressed in Treg cells, was critical for their suppressor function. The deficiency of miR-146a in Treg cells resulted in a breakdown of immunological tolerance manifested in fatal IFN-γ-dependent immune-mediated lesions in a variety of organs. This was likely due to augmented expression and activation of STAT1, a direct target of miR-146a [34]. MiR-150 and miR-223 target the STAT1 3′UTR, reduce STAT1 expression, and reduce both IFN-dependent and IFN-independent STAT1-mediated signaling [35]. MiR-27a and miR-220c were regulated by STAT1. In the meanwhile, miR-27a and miR-220c can in turn target the 3′UTRs of STAT1 [31]. Therefore, miR-200c may regulate PD-L1 expression by target IFN-γ and STAT1.

#### 2.2.4. MicroRNAs Regulating IRF1 Expression

Luciferase assays demonstrated that IRF1 was directly regulated by miR-23b and miR-383. MiR-23b over-expression significantly decreased IRF1 mRNA levels [36]. Both IRF1 protein and IRF1 mRNA expressions were significantly decreased in miR-383-transfected NT2 (testicular embryonal carcinoma) cells [37].

#### 2.2.5. MicroRNAs Regulating PTEN Expression

Like PD-L1, many microRNAs have been shown to inhibit PTEN expression by directly binding to PTEN 3′UTR (Table 1).

MiR-10a was upregulated in non-small-cell lung carcinoma (NSCLC) compared with corresponding normal tissues. Furthermore, over-expression of miR-10a promoted NSCLC cell proliferation, migration and invasion, suggesting that miR-10a contributes to NSCLC by targeting PTEN [38]. MiR-19a and miR-19b were upregulated in gastric cancer cells and decreased the sensitivity of gastric cancer cells to anticancer drugs by inhibiting the expression of PTEN [39]. PTEN expression was inhibited by miR-20b and miR-21 through binding with the 3′UTR of PTEN mRNA in colorectal cancer (CRC), resulting in PD-L1 over-expression [40]. MiR-26a reduced the expression level of PTEN in A549, SK-MES-1, and H661 cells. When miR-26a was knockdown in H661 cells, the expression level of PTEN increased [41]. MiR-92a was over-expressed in colorectal cancer cell lines. Up-regulation of miR-92a decreased the expression of PTEN [42]. MiR-106b has been shown to target PD-L1. Meanwhile, miR-106 had been found to inhibit PTEN through directly binding to its 3′UTR [43]. MiR-205 could inhibit expression of PTEN by directly targeting the 3′UTR of PTEN gene in nasopharyngeal carcinoma (NPC) [44]. MiR-214 induces cell survival through targeting the 3′UTR of the PTEN, which leads to down-regulation of PTEN protein and activation of AKT pathway [45]. MiR-221 and miR-222 were discovered to induce cell growth and cell cycle progression via direct modulation of PTEN expression [46]. Expression of miR-301a was markedly elevated in breast cancer. MiR-301a promoted breast cancer invasion via directly targeting the 3′UTR of PTEN gene and subsequent down-regulation of PTEN [47]. MiR-494 in myeloid-derived suppressor cells (MDSCs) was up-regulated and played a critical role in promoting tumor growth and metastasis by combining to the 3′UTR of PTEN and inhibiting PTEN [48].

#### 2.2.6. MicroRNAs Regulatingm TOR Expression

Four microRNAs (miR-100, miR-101, miR-199a-3p, and miR-497) have been found to inhibit mTOR by directly combining to its 3′UTR using luciferase assays.

MiR-100 could inhibit bladder cancer cell growth and colony formation by targeting mTOR [49]. Overexpression of miR-101 significantly decreased the expression of mTOR at both mRNA and protein levels in Saos-2 cells [50]. MiR-199a-3p could inhibit endometrioid adenocarcinoma (EEC) cell proliferation through binding to the 3′-UTR of mTOR [51]. Low miR-497 expression levels were associated with chemo-resistant phonotype of ovarian cancer. MiR-497 could suppress mTOR protein expressions through the binding of mTOR 3′UTR. Downregulation of miR-497 contributed to high levels of mTOR [52].

#### 2.2.7. MicroRNAs Regulating Eukaryotic Translation Initiation Factor 4B (EIF4B) Expression

EIF4B integrates the signals from the PI3K/AKT/mTOR pathway [62] and is directly regulated by miR-150 and miR-216a. MiR-150 could suppress EIF4B protein expressions through binding to the 3′UTR of EIF4B. Downregulation of miR-497 contributed to high levels of EIF4B [53]. Over-expression of miR-216a suppresses NSCLC cell growth, metastasis, and enhances cisplatin-induced cell growth inhibition and apoptosis by directly targeting EIF4B [54].

#### 2.2.8. MicroRNAs Regulating SHP2 Expression

As with IFNGR, only one microRNA was reported to regulate SHP2 expression. MiR-204 could inhibit SHP2 via combining to the 3′UTR of SHP2 using luciferase assays [55].

#### 2.2.9. MicroRNAs Regulating c-Fos Protein (c-Fos) Expression

Finally, six microRNAs (miR-101, miR-139-5p, miR-155, miR-181a, miR-181b, and miR-490-5p) have been shown that involved in the regulation of c-Fos.

MiR-101 was downregulated and acted as a tumor suppressor in osteosarcoma (OS) cells via targeting c-Fos [56]. MiR-139-5p suppressed c-Fos transcription through binding to the 3′-UTR of c-Fos in primary biliary cholangitis [57]. C-Fos mRNA levels were found to decrease in a manner that was closely correlated with increased miR-155 expression during the activation of human monocyte-derived DCs (Mo-DCs). MiR-155 regulates both the stability and translation of c-Fos mRNA [58]. MiR-181a targeted the 3′-UTR of c-Fos, abundance of miR-181a reduced c-Fos protein, whereas inhibition of miR-181a increased c-Fos protein in bone marrow-derived DCs (BMDCs) [59]. MiR-181b regulated FOS expression by directly targeting the binding site within the 3′-UTR in malignant gliomas [60]. MiR-490-5p was downregulated in human bladder cancer tissue and cell lines compared to normal adjacent tissue and a non-malignant cell line. MiR-490-5p is a novel tumor suppressor of bladder cancer cell proliferation through targeting c-Fos [61].

## 3. Conclusions

PD-1/PD-L1 immune checkpoint plays an important role in tumor immune escape and the formation of tumor microenvironment. MicroRNAs have shown a new avenue in the understanding of the molecular regulatory mechanisms involved in PD-1/PD-L1 signaling pathway. MicroRNAs are able to modulate multiple signaling pathways and regulatory networks. Even if the change of microRNAs expression is subtle, it may cause significant changes in PD-1/PD-L1 signaling pathway. Manipulation of PD-1/PD-L1 expression is not the only option for microRNAs to regulate PD-1/PD-L1 pathway. MicroRNAs regulating the transport or downstream targets of PD-1/PD-L1 may also have a significant impact on PD-1/PD-L1 pathway. For example, the PD-1downstream molecule Zeta-chain-associated protein kinase 70 (ZAP70) is regulated by miR-631 [63]. In this review, we focused on these microRNAs regulating PD-1/PD-L1 expression and demonstrated that 49 microRNAs target the 3′-UTR of either PD-1/PD-L1 themselves or their upstream genes. Among them, five microRNA (miR-101, miR-106b, miR-181a, miR-150, and miR-200c) have two target mRNA. Currently little is known about the correlation between these microRNAs and the prognostic roles of PD-L1 or the therapeutic effects of the PD-1/PD-L1 antibodies. Significant advances have been made for employing microRNAs as useful diagnostic targets. It is possible that in addition for further understanding the molecular regulatory network of PD-1/PD-L1, these microRNAs can also be potential useful biomarkers for prognosis of PD-1/PD-L1 antibody treatment.

## Figures and Tables

**Table 1:** MicroRNAs involved in PD-1/PD-L1 signaling pathway and their respective target genes.

Target mRNA	miRNA	Host	Reference
**PD-1**	miR-28	melanoma	Li et al. [17]
	miR-138	glioblastoma	Wei et al. [18]
	miR-4717	HCC	Zhang et al. [19]
**PD-L1**	miR-15a	MPM	Kao et al. [20]
	miR-15b	MPM	Kao et al. [20]
	miR-16	MPM	Kao et al. [20]
	miR-34a	AML	Wang et al. [21]
	miR-93	MSC	Cioffi et al. [22]
	miR-106b	MSC	Cioffi et al. [22]
	miR-138-5p	CRC	Zhao et al. [23]
	miR-142-5p	PC	Jia et al. [24]
	miR-152	GC	Wang et al. [25]
	miR-193a-3p	MPM	Kao et al. [20]
	miR-200	GC	Chen et al. [26]
	miR-324-5p	Treg	Holla et al. [27]
	miR-338-5p	Treg	Holla et al. [27]
	miR-424	OC	Xu et al. [28]
	miR-513	Cholangiocytes	Gong et al. [29]
**IFN-γ**	miR-181a	aGVHD	Sang et al. [30]
	miR-24-2	HeLa	Wang et al. [31]
	miR-200c	HeLa	Wang et al. [31]
**IFNGR**	miR-378	CL	Ma et al. [32]
**STAT1**	miR-27a	HeLa	Wang et al. [31]
	miR-145	CC	Gregersen et al. [33]
	miR-146a	Treg	Lu et al. [34]
	miR-150	ATL	Moles et al. [35]
	miR-223	ATL	Moles et al. [35]
	miR-200c	HeLa	Wang et al. [31]
**IRF1**	miR-23b	DF-1	Li et al. [36]
	miR-383	TEC	Lian et al. [37]
**PTEN**	miR-10a	NSCLC	Yu et al. [38]
	miR-19a	GC	Wang et al. [39]
	miR-19b	GC	Wang et al. [39]
	miR-20b	CRC	Zhu et al. [40]
	miR-21	CRC	Zhu et al. [40]
	miR-26a	LC	Liu et al. [41]
	miR-92a	CRC	Zhang et al. [42]
	miR-106b	GC	Yang et al. [43]
	miR-205	NPC	Qu et al. [44]
	miR-214	OC	Yang et al. [45]
	miR-221	GC	Zhang et al. [46]
	miR-222	GC	Zhang et al. [46]
	miR-301a	BC	Ma et al. [47]
	miR-494	MDSCs	Liu et al. [48]
**MTOR**	miR-100	BLC	Xu et al. [49]
	miR-101	OTC	Lin et al. [50]
	miR-199a-3p	EEC	Wu et al. [51]
	miR-497	OC	Xu et al. [52]
**EIF4B**	miR-150	HSC	Fang et al. [53]
	miR-216a	NSCLC	Wang et al. [54]
**SHP2**	miR-204	PASMCs	Courboulin et al. [55]
**FOS**	miR-101	OS	Wang et al. [56]
	miR-139-5p	PBC	Katsumi1 et al. [57]
	miR-155	DCs	Dunand-Sauthier et al. [58]
	miR-181a	DCs	Wu et al. [59]
	miR-181b	MG	Tao et al. [60]
	miR-490-5p	BLC	Li et al. [61]

aGVHD = Acute graft-versus-host; CL = corpus luteum; CC = colon cancer; Treg = Foxp3 + regulatory T Cell; ATL = adult T cell leukemia/lymphoma; TEC = testicular embryonal carcinoma; NSCLC = non-small cell lung cancer; GC = gastric cancer; CRC = colorectal cancer; LC = lung cancer; NPC = nasopharyngeal carcinoma; OC = ovarian cancer; BC = breast cancer; MDSCs = myeloid-derived suppressor cells; BLC = bladder cancer; OTC = osteosarcoma cells; EEC = endometrial cancer cell; HSC = hematopoietic stem cells; PASMCs = pulmonary artery smooth muscle cells; DCs = dendritic cell; MPM = malignant pleural mesothelioma; AML = acute myeloid leukemia; MSC = mesenchymal stem cells; PC = pancreatic cancer; HCC = hepatocellular carcinoma. OS = osteosarcoma; PBC = primary biliary cholangitis; MG = malignant gliomas.

## References

[1] Dong H., Strome S.E., Salomao D.R., Tamura H., Hirano F., Flies D.B., Roche P.C., Lu J., Zhu G., Tamada K. (2002). Tumor-associated B7-H1 promotes T-cell apoptosis: A potential mechanism of immune evasion. Nat. Med..

[2] Hirano F., Kaneko K., Tamura H., Dong H., Wang S., Ichikawa M., Rietz C., Flies D.B., Lau J.S., Zhu G. (2005). Blockade of B7-H1 and PD-1 by Monoclonal Antibodies Potentiates Cancer Therapeutic Immunity. Cancer Res..

[3] Eppihimer M.J., Gunn J., Freeman G.J., Greenfield E.A., Chernova T., Erickson J., Leonard J.P. (2002). Expression and Regulation of the PD-L1 Immunoinhibitory Molecule on Microvascular Endothelial Cells. Microcirculation.

[4] Mühlbauer M., Fleck M., Schütz C., Weiss T., Froh M., Blank C., Schölmerich J., Hellerbrand C. (2006). PD-L1 is induced in hepatocytes by viral infection and by interferon-α and -γ and mediates T cell apoptosis. J. Hepatol..

[5] Liu J., Hamrouni A., Wolowiec D., Coiteux V., Kuliczkowski K., Hetuin D., Saudemont A., Quesnel B. (2007). Plasma cells from multiple myeloma patients express B7-H1 (PD-L1) and increase expression after stimulation with IFN-γ and TLR ligands via a MyD88-, TRAF6-, and MEK-dependent pathway. Blood.

[6] Gu X., Wang Y., Xiang J., Chen Z., Wang L., Lu L., Qian S. (2013). Interferon-γ triggers hepatic stellate cell-mediated immune regulation through MEK/ERK signaling pathway. Clin. Dev. Immunol..

[7] Parsa A.T., Waldron J.S., Panner A., Crane C.A., Parney I.F., Barry J.J., Cachola K.E., Murray J.C., Tihan T., Jensen M.C. (2007). Loss of tumor suppressor PTEN function increases B7-H1 expression and immunoresistance in glioma. Nat. Med..

[8] Taniguchi T., Ogasawara K., Takaoka A., Tanaka N. (2001). IRF family of transcription factors as regulators of host defense. Annu. Rev. Immunol..

[9] Collins F.S., Lander E.S., Rogers J., Waterson R.H. (2003). Finishing the euchromatic sequence of the human genome International Human Genome Sequencing Consortium Nature 2004 431 931 45. Nature.

[10] Ambros V. (2004). The functions of animal microRNAs. Nature.

[11] Lee Y., Ahn C., Han J., Choi H., Kim J., Yim J., Lee J., Provost P., Rådmark O., Kim S. (2003). The nuclear RNase III Drosha initiates microRNA processing. Nature.

[12] Lee Y., Jeon K., Lee J.T., Kim S., Kim V.N. (2002). MicroRNA maturation: Stepwise processing and subcellular localization. EMBO J..

[13] Lund E., Güttinger S., Calado A., Dahlberg J.E., Kutay U. (2004). Nuclear Export of MicroRNA Precursors. Science.

[14] Finnegan E.J., Matzke M.A. (2003). The small RNA world. J. Cell Sci..

[15] Pichler M., Calin G.A. (2015). MicroRNAs in cancer: From developmental genes in worms to their clinical application inpatients. Br. J. Cancer.

[16] Platanias L.C. (2005). Mechanisms of type-I- and type-II-interferon-mediated signalling. Nat. Rev. Immunol..

[17] Li Q., Johnston N., Zheng X., Wang H., Zhang X., Gao D., Min W. (2016). MiR-28 modulates exhaustive differentiation of T cells through silencing programmed cell death-1 and regulating cytokine secretion. Oncotarget.

[18] Wei J., Nduom E., Kong L.Y., Wang F., Xu S., Gabrusiewicz K., Alum A., Fuller G., Calin G., Heimberger A.B. (2013). MiR-138 exerts anti-glioma efficacy by targeting immune checkpoints. J. Immunother. Cancer.

[19] Zhang G., Na L., Zhu L., Zhu Q., Fang L., Yang C., Han Q., Yi L., Zhou Z., Liu Z. (2015). MicroRNA-4717 differentially interacts with its polymorphic target in the PD1 3′ untranslated region: A mechanism for regulating PD-1 expression and function in HBV-associated liver diseases. Oncotarget.

[20] Kao S.C., Cheng Y.Y., Williams M., Kirschner M.B., Madore J., Lum T., Sarun K.H., Linton A., Mccaughan B., Klebe S. (2017). Tumour suppressor microRNAs contribute to the regulation of PD-L1 expression in malignant pleural mesothelioma. J. Thorac. Oncol..

[21] Wang X., Li J., Dong K., Lin F., Long M., Ouyang Y., Wei J., Chen X., Weng Y., He T. (2015). Tumor suppressor miR-34a targets PD-L1 and functions as a potential immunotherapeutic target in acute myeloid leukemia. Cell Signal..

[22] Cioffi M., Trabulo S.M., Vallespinos M., Raj D., Kheir T.B., Lin M.L., Begum J., Baker A.M., Amgheib A., Saif J. (2017). The miR-25-93-106b cluster regulates tumor metastasis and immune evasion via modulation of CXCL12 and PD-L1. Oncotarget.

[23] Zhao L., Yu H., Yi S., Peng X., Peng S., Xiao Z., Liu R., Tang A., Li X., Liu F. (2016). The tumor suppressor miR-138-5p targets PD-L1 in colorectal cancer. Oncotarget.

[24] Jia L., Xi Q., Wang H., Zhang Z., Liu H., Cheng Y., Guo X., Zhang J., Zhang Q., Zhang L. (2017). MiR-142-5p regulates tumor cell PD-L1 expression and enhances anti-tumor immunity. Biochem. Biophys. Res. Commun..

[25] Wang Y., Di W., Xie G., Yin Y., Zhao E., Tao K., Li R. (2017). MicroRNA-152 regulates immune response via targeting B7-H1 in gastric carcinoma. Oncotarget.

[26] Chen L., Gibbons D.L., Goswami S., Cortez M.A., Ahn Y.H., Byers L.A., Zhang X., Yi X., Dwyer D., Lin W. (2014). Metastasis is regulated via microRNA-200/ZEB1 axis control of tumour cell PD-L1 expression and intratumoral immunosuppression. Nat. Commun..

[27] Holla S., Stephenvictor E., Prakhar P., Sharma M., Saha C., Udupa V., Kaveri S.V., Bayry J. (2016). Mycobacteria-responsive sonic hedgehog signaling mediates programmed death-ligand 1- and prostaglandin E2-induced regulatory T cell expansion. Sci. Rep..

[28] Xu S., Tao Z., Hai B., Liang H., Shi Y., Wang T., Song W., Chen Y., Ouyang J., Chen J. (2016). MiR-424(322) reverses chemoresistance via T-cell immune response activation by blocking the PD-L1 immune checkpoint. Nat. Commun..

[29] Gong A.Y., Zhou R., Hu G., Liu J., Sosnowska D., Drescher K.M., Dong H., Chen X.M. (2010). Cryptosporidium parvum Induces B7-H1 Expression in Cholangiocytes by Downregulating MicroRNA-513. J. Infect. Dis..

[30] Sang W., Zhang C., Zhang D., Wang Y., Sun C., Niu M., Sun X., Zhou C., Zeng L., Pan B. (2015). MicroRNA-181a, a Potential Diagnosis Marker, Alleviates Acute Graft Versus Host Disease by Regulating IFN-γ Production. Am. J. Hematol..

[31] Wang G., Wang Y., Teng M., Zhang D., Li L., Liu Y. (2010). Signal Transducers and Activators of Transcription-1 (STAT1) Regulates microRNA Transcription in Interferon γ-Stimulated HeLa Cells. PLoS ONE.

[32] Ma T., Jiang H., Gao Y., Zhao Y., Dai L., Xiong Q., Xu Y., Zhao Z., Zhang J. (2011). Microarray analysis of differentially expressed microRNAs in non-regressed and regressed bovine corpus luteum tissue; microRNA-378 may suppress luteal cell apoptosis by targeting the interferon γ receptor 1 gene. J. Appl. Genet..

[33] Gregersen L.H., Jacobsen A.B., Frankel L.B., Wen J., Krogh A., Lund A.H. (2010). MicroRNA-145 Targets YES and STAT1 in Colon Cancer Cells. PLoS ONE.

[34] Lu L.F., Boldin M.P., Chaudhry A., Lin L.L., Taganov K.D., Hanada T., Yoshimura A., Baltimore D., Rudensky A.Y. (2010). Function of miR-146a in controlling Treg cell-mediated regulation of Th1 responses. Cell.

[35] Moles R., Bellon M., Nicot C. (2015). STAT1: A Novel Target of miR-150 and miR-223 Is Involved in the Proliferation of HTLV-I-Transformed and ATL Cells. Neoplasia.

[36] Li Z., Chen B., Feng M., Ouyang H., Zheng M., Ye Q., Nie Q., Zhang X. (2015). MicroRNA-23b Promotes Avian Leukosis Virus Subgroup J (ALV-J) Replication by Targeting IRF1. Sci. Rep..

[37] Lian J., Tian H., Liu L., Zhang X.S., Li W.Q., Deng Y.M., Yao G.D., Yin M.M., Sun F. (2010). Downregulation of microRNA-383 is associated with male infertility and promotes testicular embryonal carcinoma cell proliferation by targeting IRF1. Cell Death Dis..

[38] Yu T., Liu L., Li J., Yan M., Lin H., Liu Y., Chu D., Tu H., Gu A., Yao M. (2015). MiRNA-10a is upregulated in NSCLC and may promote cancer by targeting PTEN. Oncotarget.

[39] Wang F., Li T., Zhang B., Li H., Wu Q., Yang L., Nie Y., Wu K., Shi Y., Fan D. (2013). MicroRNA-19a/b regulates multidrug resistance in human gastric cancer cells by targeting PTEN. Biochem. Biophys. Res. Commun..

[40] Zhu J., Chen L., Zou L., Yang P., Wu R., Mao Y., Zhou H., Li R., Wang K., Wang W. (2014). MiR-20b, -21, and -130b inhibit PTEN expression resulting in B7-H1 over-expression in advanced colorectal cancer. Hum. Immunol..

[41] Liu B., Wu X., Liu B., Wang C., Liu Y., Zhou Q., Xu K. (2012). MiR-26a enhances metastasis potential of lung cancer cells via AKT pathway by targeting PTEN. Biochim. Biophys. Acta.

[42] Zhang G., Zhou H., Xiao H., Liu Z., Tian H., Zhou T. (2014). MicroRNA-92a functions as an oncogene in colorectal cancer by targeting PTEN. Dig. Dis. Sci..

[43] Yang T.S., Yang X.H., Chen X., Wang X.D., Hua J., Zhou D.L., Zhou B., Song Z.S. (2014). MicroRNA-106b in cancer-associated fibroblasts from gastric cancer promotes cell migration and invasion by targeting PTEN. FEBS Lett..

[44] Qu C., Liang Z., Huang J., Zhao R., Su C., Wang S., Wang X., Zhang R., Lee M.H., Yang H. (2012). MiR-205 determines the radioresistance of human nasopharyngeal carcinoma by directly targeting PTEN. Cell Cycle.

[45] Yang H., Kong W., He L., Zhao J.J., O’Donnell J.D., Wang J., Wenham R.M., Coppola D., Kruk P.A., Nicosia S.V. (2008). MicroRNA expression profiling in human ovarian cancer: MiR-214 induces cell survival and cisplatin resistance by targeting PTEN. Cancer Res..

[46] Zhang C., Han L., Zhang A., Yue X., Wang G., Jia Z., Pu P., Zhang Q., Kang C. (2010). MicroRNA-221 and microRNA-222 regulate gastric carcinoma cell proliferation and radioresistance by targeting PTEN. BMC Cancer.

[47] Ma F., Zhang J., Zhong L., Wang L., Liu Y., Wang Y., Peng L., Guo B. (2014). Upregulated microRNA-301a in breast cancer promotes tumor metastasis by targeting PTEN and activating Wnt/β-catenin signaling. Gene.

[48] Liu Y., Lai L., Chen Q., Song Y., Xu S., Ma F., Wang X., Wang J., Yu H., Cao X. (2012). MicroRNA-494 is required for the accumulation and functions of tumor-expanded myeloid-derived suppressor cells via targeting of PTEN. J. Immunol..

[49] Xu C., Zeng Q., Xu W., Jiao L., Chen Y., Zhang Z., Wu C., Jin T., Pan A., Wei R. (2013). MiRNA-100 inhibits human bladder urothelial carcinogenesis by directly targeting mTOR. Mol. Cancer Ther..

[50] Lin S., Shao N.N., Fan L., Ma X.C., Pu F.F., Shao Z.W. (2014). Effect of microRNA-101 on proliferation and apoptosis of human osteosarcoma cells by targeting mTOR. J. Huazhong Univ. Sci. Technol. Med. Sci..

[51] Wu D., Huang H.J., He C.N., Wang K.Y. (2013). MicroRNA-199a-3p regulates endometrial cancer cell proliferation by targeting mammalian target of rapamycin (mTOR). Int. J. Gynecol. Cancer.

[52] Xu S., Fu G.B., Tao Z., Ouyang J., Kong F., Jiang B.H., Wan X., Chen K. (2015). MiR-497 decreases cisplatin resistance in ovarian cancer cells by targeting mTOR/P70S6K1. Oncotarget.

[53] Fang Z.H., Wang S.L., Zhao J.T., Lin Z.J., Chen L.Y., Su R., Xie S.T., Bing Z.C., Xu B. (2016). MiR-150 exerts antileukemia activityin vitroandin vivothrough regulating genes in multiple pathways. Cell Death Dis..

[54] Wang R.T., Xu M., Xu C.X., Song Z.G., Jin H. (2014). Decreased expression of miR216a contributes to non-small-cell lung cancer progression. Clin. Cancer Res..

[55] Courboulin A., Paulin R., Giguère N.J., Saksouk N., Perreault T., Meloche J., Paquet E.R., Biardel S., Provencher S., Côté J. (2011). Role for miR-204 in human pulmonary arterial hypertension. J. Exp. Med..

[56] Wang Z., Rongzhen H.E., Xia H., Wei Y.U., Song W.U. (2016). MicroRNA-101 has a suppressive role in osteosarcoma cells through the targeting of c-FOS. Exp. Ther. Med..

[57] Katsumi T., Ninomiya M., Nishina T., Mizuno K., Tomita K., Haga H., Okumoto K., Saito T., Shimosegawa T., Ueno Y. (2016). MiR-139-5p is associated with inflammatory regulation through c-FOS suppression, and contributes to the progression of primary biliary cholangitis. Lab. Investig..

[58] Dunand-Sauthier I., Santiago-Raber M.L., Capponi L., Vejnar C.E., Schaad O., Irla M., Seguín-Estévez Q., Descombes P., Zdobnov E.M., Acha-Orbea H. (2011). Silencing of c-Fos expression by microRNA-155 is critical for dendritic cell maturation and function. Blood.

[59] Wu C., Gong Y., Yuan J., Zhang W., Zhao G., LI H., Sun A., Hu K., Zou Y., Ge J. (2012). MicroRNA-181a represses ox-LDL-stimulated inflammatory response in dendritic cell by targeting c-Fos. J. Lipid Res..

[60] Tao T., Wang Y., Luo H., Yao L., Wang L., Wang J., Zhang J., Wang H., Shi Y., Yin Y. (2013). Involvement of FOS-mediated miR-181b/miR-21 signalling in the progression of malignant gliomas. Eur. J. Cancer.

[61] Li S., Xu X., Xu X., Hu Z., Wu J., Zhu Y., Chen H., Mao Y., Lin Y., Luo J. (2013). MicroRNA-490-5p inhibits proliferation of bladder cancer by targeting c-Fos. Biochem. Biophys. Res. Commun..

[62] Chen K., Yang J., Li J., Wang X., Chen Y., Huang S., Chen J.L. (2016). EIF4B is a convergent target and critical effector of oncogenic Pim and PI3K/Akt/mTOR signaling pathways in Abl transformants. Oncotarget.

[63] Fu D., Liu B., Zang L.E., Jiang H. (2015). MiR-631/ZAP70: A novel axis in the migration and invasion of prostate cancer cells. Biochem. Biophys. Res. Commun..

